# Tyrosine Kinase Inhibitors for Gastrointestinal Stromal Tumor After Imatinib Resistance

**DOI:** 10.3390/pharmaceutics17070923

**Published:** 2025-07-17

**Authors:** Xian-Hao Xiao, Qian-Shi Zhang, Ji-Yuan Hu, Yin-Xu Zhang, He Song

**Affiliations:** 1 Department of Gastrointestinal Surgery, The First Hospital of China Medical University, Shenyang 110001, China; daoseeker30@163.com; 2 Department of Gastrointestinal Surgery, The Second Affiliated Hospital of Dalian Medical University, Dalian 116023, China; zhangqs@dmu.edu.cn; 3 Department of Urology, The First Hospital of China Medical University, Shenyang 110001, China; humark1999@163.com; 4 Department of General Surgery, The First Affiliated Hospital of Jinzhou Medical University, Jinzhou 121001, China

**Keywords:** gastrointestinal stromal tumor, tyrosine kinase inhibitor, imatinib resistance, efficacy, safety

## Abstract

Gastrointestinal stromal tumors (GISTs) are the most common mesenchymal tumors of the gastrointestinal tract, primarily driven by activating mutations in KIT (CD117) and platelet-derived growth factor receptor alpha (PDGFRA). The introduction of tyrosine kinase inhibitors (TKIs), especially imatinib, has significantly transformed GIST treatment. However, the emergence of both primary and secondary resistance to imatinib presents ongoing therapeutic challenges. This review comprehensively explores the mechanisms underlying imatinib resistance and evaluates subsequent TKI therapies. Sunitinib, regorafenib, and ripretinib are currently approved as standard second-, third-, and fourth-line therapies, each demonstrating efficacy against distinct mutational profiles. Avapritinib, notably effective against PDGFRA D842V mutations, represents a milestone for previously untreatable subgroups. Several alternative agents—such as nilotinib, masitinib, sorafenib, dovitinib, pazopanib, and ponatinib—have shown varying degrees of success in refractory cases or specific genotypes. Investigational compounds, including crenolanib, bezuclastinib, famitinib, motesanib, midostaurin, IDRX-42, and olverembatinib, are under development to address resistant or wild-type GISTs. Despite progress, long-term efficacy remains limited due to evolving resistance. Future strategies include precision medicine approaches such as ctDNA-guided therapy, rational drug combinations, and novel drug delivery systems to optimize bioavailability and reduce toxicity. Ongoing research will be crucial for refining treatment sequencing and expanding therapeutic options, especially for rare GIST subtypes.

## 1. Introduction

Gastrointestinal stromal tumors (GISTs) are the most prevalent mesenchymal neoplasms of the gastrointestinal tract, with a global incidence of approximately 1 per 100,000 individuals annually [1,2]. Arising from the precursors of the interstitial cells of Cajal (ICC), GISTs are predominantly driven by activating mutations in the KIT (CD117) or platelet-derived growth factor receptor alpha (PDGFRA) genes [3,4,5,6]. KIT and PDGFRA are two genes that encode receptor tyrosine kinases, proteins that sit on the surface of cells and play critical roles in cell signaling, growth, and survival. GISTs are insensitive to conventional chemotherapy agents [7]. However, the advent of imatinib mesylate, a representative tyrosine kinase inhibitor (TKI), has revolutionized the treatment of advanced GISTs by specifically targeting these aberrant kinases, resulting in substantial clinical benefit and improved survival [8,9]. Despite its initial efficacy, imatinib resistance—both primary and secondary—inevitably emerges in a significant proportion of patients, posing a major therapeutic challenge [10].

Primary resistance typically occurs in tumors harboring imatinib-insensitive mutations, such as PDGFRA D842V, while secondary resistance often arises due to additional mutations within the KIT gene that interfere with imatinib binding [11,12,13]. This has necessitated the development and implementation of second- and over-second-line TKIs designed to overcome resistance mechanisms and target a broader spectrum of mutant kinases.

In recent years, several next-generation TKIs, including sunitinib, regorafenib, and ripretinib, have demonstrated efficacy in imatinib-refractory GIST, each with distinct kinase inhibition profiles and clinical utility. Furthermore, novel agents and combination strategies are also under investigation, aiming to overcome resistance more effectively and improve long-term disease control.

This review provides an overview of the current landscape of TKIs used following imatinib resistance in GISTs, highlighting their mechanisms of action, clinical efficacy, resistance patterns, and future therapeutic directions.

## 2. Relevance and Distinction from Existing Literature

GISTs continue to be a focal point of oncological research due to the complexities associated with TKI resistance and the emergence of novel therapeutic agents. Recent studies have delved into various aspects of GIST management. The 2023 GEIS guidelines provide comprehensive updates on the management of GISTs, emphasizing the roles of approved TKIs and the importance of molecular profiling in treatment decisions [14]. In addition, investigations into circulating tumor DNA (ctDNA) have highlighted its potential in monitoring disease progression and guiding therapy, offering a non-invasive method to detect resistance mutations [15]. Clinical trials, such as the AGITG ALT-GIST study, have explored alternative dosing regimens and combinations of TKIs to overcome resistance, although with varying degrees of success [16].

While these studies contribute significantly to our understanding of GISTs, our manuscript distinguishes itself by providing a comprehensive synthesis of the mechanisms underlying imatinib resistance and a detailed evaluation of both approved and investigational TKIs. We integrate recent findings on drug formulations, resistance patterns, and emerging therapies, offering a holistic view that bridges current knowledge gaps. Furthermore, our discussion on future directions, including precision medicine approaches and novel drug delivery systems, provides a forward-looking perspective not extensively covered in existing reviews.

## 3. Methods of Literature Selection

This review was based on a structured literature search conducted across several databases, including PubMed, Embase, Web of Science, and ClinicalTrials.gov, covering publications up to May 2025. The search strategy incorporated combinations of the following keywords: “gastrointestinal stromal tumor”, “GIST”, “tyrosine kinase inhibitor”, “TKI”, “imatinib resistance”, and the specific names of TKIs. The inclusion criteria were (1) peer-reviewed original research articles, clinical trials, and review articles focused on GISTs and their treatment with TKIs; (2) studies evaluating drug efficacy, resistance mechanisms, or molecular profiles; and (3) English-language articles. The exclusion criteria included (1) non-English publications and (2) editorials or commentaries without primary data or review synthesis. The final selection was based on their relevance to the review objectives, novelty, clinical applicability, and data quality. Priority was given to phase II/III clinical trials, large cohort studies, and high-impact reviews published within the last 10 years.

## 4. Imatinib Resistance

Imatinib mesylate, a selective tyrosine kinase inhibitor, has revolutionized the treatment of GISTs by targeting the constitutively activated KIT or PDGFRA receptors, which are the primary oncogenic drivers in approximately 85–90% of cases. Imatinib binds to the ATP-binding pocket of these kinases, stabilizing their inactive conformation and thereby inhibiting downstream signaling pathways such as MAPK and PI3K-AKT, which are critical for tumor cell proliferation and survival.

Resistance to imatinib in GISTs is broadly classified into primary and secondary resistance. Primary resistance (10–15%) occurs within the first six months of treatment and is commonly associated with specific molecular subtypes, such as PDGFRA D842V (5–8%), or rare KIT-negative GISTs [17,18]. These mutations inherently reduce imatinib binding affinity, rendering the drug ineffective from the outset.

The secondary mutations of KIT/PDGFRA, typically after 18–24 months of treatment, are the most common mechanisms (develops in 40–50% of patients) of imatinib resistance [19,20]. Point mutations occur frequently in the kinase ATP-binding domain or activation loop (encoded by exons 13, 14, 17, and 18) [21,22]. These mutations alter the conformation of the kinase domain and prevent imatinib from maintaining its inhibitory interaction with the receptor. In patients progressing after initial response, 60–70% harbor polyclonal secondary mutations, often involving multiple resistant subclones. In addition to point mutations, the copy number variation (CNV) of KIT genes, which leads to the re-activation of kinase activity, has also been demonstrated to contribute to imatinib resistance [23,24].

Alternative signaling pathways also play a role in the development of imatinib resistance. For instance, it was reported that IGF2 overexpression could upregulate the activity of the PI3K/AKT/mTOR pathway and promote imatinib resistance [25]. A comprehensive understanding of the abnormal status of signaling pathways in imatinib-resistant cases would contribute to the development of targeted therapies. Researchers conducted a clinical trial of a pan-FGFR kinase inhibitor in combination with imatinib in patients with imatinib-refractory advanced GISTs, where it was found that the FGF-related activation of MAPK can promote imatinib resistance [26].

Other mechanisms like abnormal epigenetic regulation, the reprogramming of metabolism, tumor heterogeneity and clonal evaluation, and other specific molecular mechanisms are less well characterized [27,28,29,30]. More studies are in needed to investigate the overall role of the mechanisms involved in drug resistance that are currently unclear. Understanding the molecular basis of resistance has been critical in guiding the development of novel agents with broader and more potent activity against resistant KIT and PDGFRA variants.

In summary, imatinib resistance in GISTs arises from both intrinsic and acquired mechanisms, most notably through secondary mutations in KIT or PDGFRA and tumor heterogeneity. These resistance pathways have provided critical insight into the molecular evolution of GISTs and have directly informed the rational design of next-line therapies. Importantly, the response to subsequent TKIs is often mutation-specific, highlighting the need for precise molecular profiling. Beyond simply identifying new molecules, researchers are increasingly focused on developing drugs that address the conformational flexibility of the KIT protein, enhance pharmacologic stability, and overcome polyclonal resistance by simultaneously targeting multiple mutant subclones.

## 5. Approved Agents

### 5.1. Sunitinib

**Figure 1:**
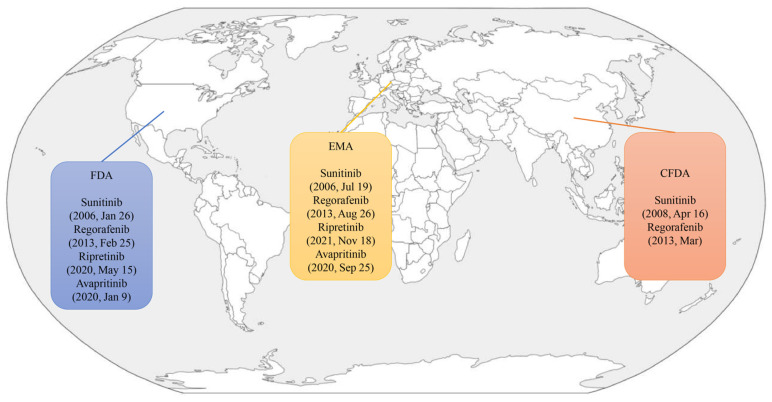
The timeline and locations of the approved TKIs.

**Figure 2:**
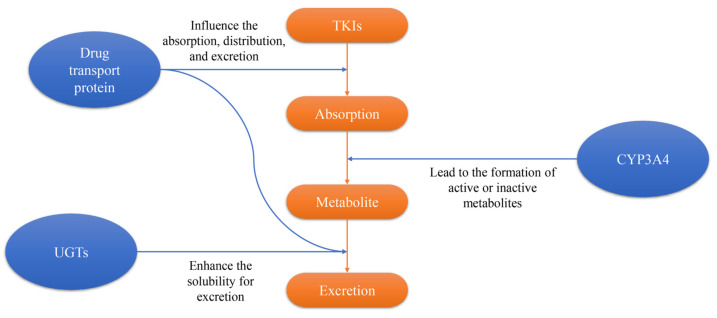
The mechanics of the drug metabolism of TKIs.

**Figure 3:**
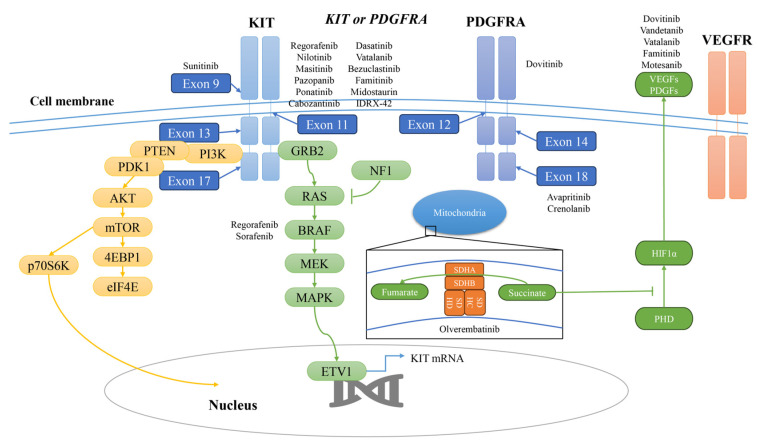
The schematic of TKI and the key pathways for GISTs.

Sunitinib malate (Sutent, SU11248; Pfizer, New York, NY, USA) is an orally active multitargeted tyrosine inhibitor that was approved as the standard second-line treatment for GISTs following progression or intolerance to imatinib by the Food and Drug Administration (FDA) in 2006 (Table 1, Figure 1) [31]. It exerts its antitumor activity by inhibiting multiple receptor tyrosine kinases, including KIT, PDGFRA, PDGFRB, VEGFRs, RET and FLT3, thereby targeting both tumor cell proliferation and angiogenesis (Figure 2 and Figure 3) [32,33,34].

**Figure 4:**
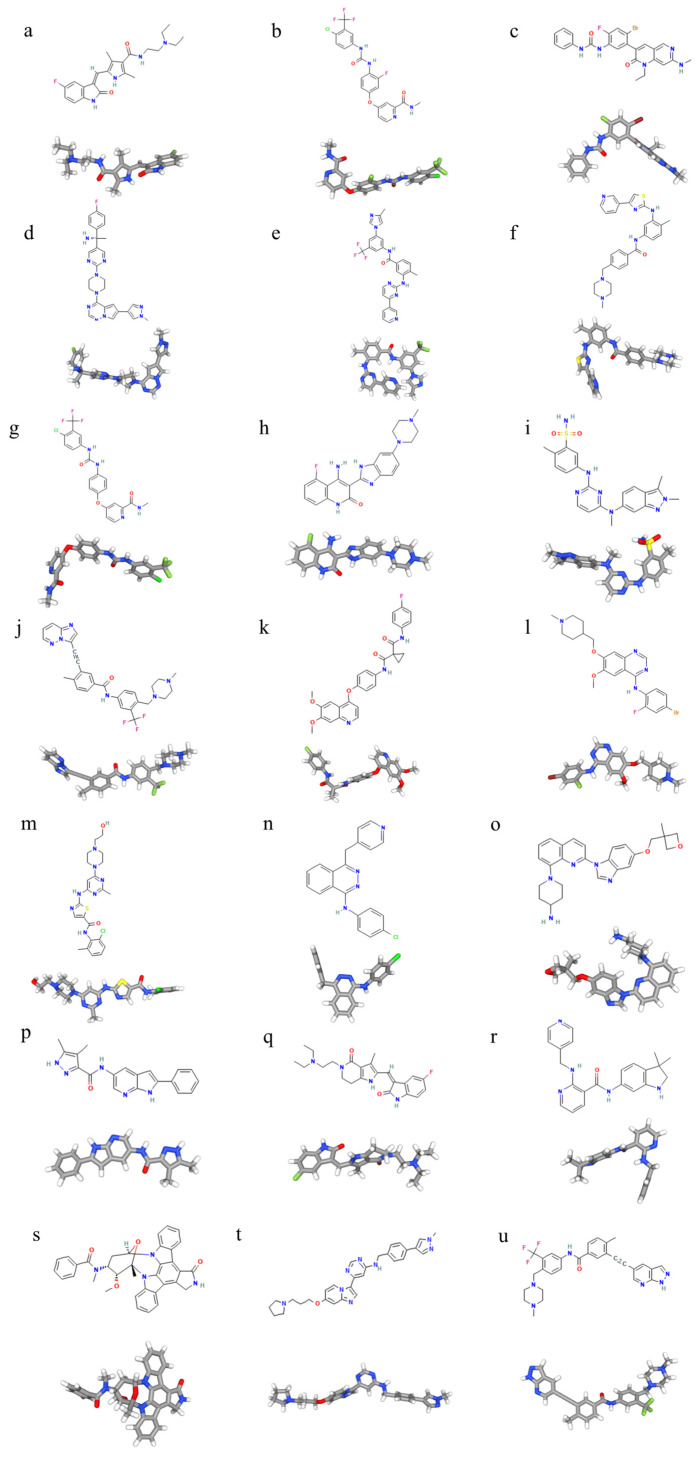
The 2D and 3D chemical structures of (**a**) sunitinib; (**b**) regorafenib; (**c**) ripretinib; (**d**) avapritinib; (**e**) nilotinib; (**f**) masitinib; (**g**) sorafenib; (**h**) dovitinib; (**i**) pazopanib; (**j**) ponatinib; (**k**) cabozantinib; (**l**) vandetanib; (**m**) dasatinib; (**n**) vatalanib; (**o**) crenolanib; (**p**) famitinib; (**q**) bezuclastinib; (**r**) motesanib; (**s**) midostaurin; (**t**) IDRX-42; and (**u**) olverembatinib.

Sunitinib has been found to have great activity against wild-type KIT and exon 9 mutations [35]. In addition, sunitinib is particularly effective in overcoming certain secondary KIT mutations that confer resistance to imatinib, especially those occurring in the ATP-binding pocket (exon 13 and 14) [36]. In comparison with imatinib, its structure and smaller molecular size affects its selectivity to the targeted size (Figure 4), contributing a broader spectrum of activity that allows for the partial suppression of the heterogeneous subclonal populations that often emerge during imatinib therapy [37].

Clinically, an intermittent dosing schedule for sunitinib of 50 mg daily for 4 weeks on and 2 weeks off was established in a phase I/II trial, although continuous dosing regimens of 37.5 mg have also been explored to improve tolerability and maintain plasma drug levels [38,39,40,41]. Sequentially, a significant improvement in progression-free survival in patients with imatinib-resistant GIST was demonstrated in a phase III randomized placebo-controlled trial, with the option to crossover on progression [31]. In the study, patients who received sunitinib exhibited an improvement in overall survival with a hazard ratio of 0.49 (*p* = 0.007) and an over fourfold increase in the time to disease progression (27.3 weeks versus 6.4 weeks, *p* < 0.0001). It was demonstrated that anemia, neutropenia, fatigue, diarrhea, skin discoloration, nausea, and anorexia were the most frequent adverse effects (AEs). AEs of any grade were reported in 168 (83%) patients, while severe AEs (grade 3+) were observed in 40 (20%) patients (Table 2). Additionally, hand–foot syndrome and occasionally hypothyreosis were also reported to be frequently associated with sunitinib administration [42].

Heinrich and coworkers observed that gene mutation types have a significant influence on the clinical activity of sunitinib [35]. The clinical benefit was observed for common primary GIST genotypes: KIT exon 9 (58%), KIT exon 11 (34%), and wild-type KIT/PDGFRA (56%); meanwhile, PFS was found to be longer for patients with the primary KIT exon 9 mutation. Rutkowski et al. also found that patients harboring KIT mutations in exon 9 respond better than those with mutations in KIT exon 11 when administered sunitinib [43]. In particular, it was reported that GISTs carrying KITAY502-3 mutations at exon 9 exhibited the highest sensitivity [44]. In an exploratory study of ctDNA analyses from the phase III INTRIGUE trial, ctDNA was validated to be a potential biomarker for the prediction of the clinical sensitivity to sunitinib [45]. Based on this discovery, a Phase III open-label study of ripretinib versus sunitinib in patients with the gene/mutations of KIT exon 11 + 17/18 detected by ctDNA is ongoing [46].

Detailed pharmacokinetic (PK) data indicate that sunitinib has moderate bioavailability (approximately 41–58%), a Tmax between 6 and 12 h, and very high protein binding (around 95%). It is extensively distributed, with a very large volume of distribution (about 2200 L), and is primarily metabolized by CYP3A4 to its active metabolite. The parent compound has a half-life of 40–60 h while the metabolite’s half-life extends to approximately 80–100 h, and clearance is in the range of roughly 37 L/h. Because its metabolism is heavily dependent on CYP3A4, concomitant administration with strong inhibitors or inducers of CYP3A4 can significantly alter its plasma exposure, necessitating dosing adjustments for safety and efficacy [47].

Despite its efficacy, resistance to sunitinib eventually develops in most patients, often due to additional KIT mutations in the activation loop (exons 17 and 18), necessitating further therapeutic strategies. Nevertheless, sunitinib remains a cornerstone in the sequential management of GISTs after imatinib failure.

### 5.2. Regorafenib

Regorafenib (Stivarga; Bayer) is an orally administered multi-kinase inhibitor that has emerged as a vital treatment option for patients with advanced GIST after the failure of first- and second-line therapies [48,49,50]. Its approval was primarily based on data from pivotal studies, most notably the GRID trial, which demonstrated that regorafenib significantly prolonged PFS compared to the placebo in imatinib- and sunitinib-refractory GIST patients [51].

Its antitumor effects are mostly based on its potent inhibition of a broad spectrum of receptor tyrosine kinases involved in tumor cell proliferation, angiogenesis, and the tumor microenvironment. It targets key kinases including KIT, PDGFRA, PDGFRB, VEGFR1-3, TIE2, RET, BRAF (including the V600E mutant form), RAF1, and FGFR, contributing to its multifaceted mechanism of action [52]. Through dual anti-proliferative and anti-angiogenic effects, regorafenib not only directly impedes tumor growth but also disrupts the formation of new blood vessels necessary for tumor progression.

Regorafenib is indicated for the management of advanced GISTs in patients whose disease has progressed despite prior treatments with imatinib and sunitinib. In clinical practice, it became the standard third-line therapy in 2013. The Phase III GRID trial (199 patients enrolled) showed that regorafenib improved the median PFS to approximately 4.8 months compared with 0.9 months in the placebo group (HR = 0.27, *p* < 0.0001) in the context of the best supportive care, highlighting a marked delay in disease progression for a highly refractory patient population. Despite the modest objective response rates, with only a small proportion of patients achieving partial responses (4.5%), regorafenib is often associated with a high disease control rate (including prolonged stabilization of disease, 71.4%), which translates into meaningful clinical benefits regarding symptom management and quality of life. Intriguingly, a phase II study has verified that regorafenib is also active in the context of second-line treatment in patients with imatinib-resistant GIST, and that secondary mutation types in KIT can be a predictor for the efficacy of regorafenib [53]. Recently, REGISTRI, a phase II trial, studied the use of regorafenib in the first-line treatment of KIT/PDFGRA wild-type metastatic GISTs [54]. Within this fractional heterogeneous population, regorafenib activity compared favorably with other tyrosine kinase inhibitors, especially in the SDH-deficient GIST subset with a 11-month PFS. Based on RECIST criteria and Choi criteria, 13% and 57% of patients reached a partial response, while 87% and 43% of patients had stable disease, respectively. Both of the forementioned trials suggested that regorafenib could be considered an upfront therapy in the specific gene mutation context.

The approved dosing regimen for regorafenib in advanced GISTs is 160 mg once daily for 21 days in a 28-day cycle. However, due to its toxicity profile (61 [92.4%] with any grade of AE and 41 [61.4%] with severe AEs), which commonly includes adverse events such as hand–foot syndrome, hypertension, diarrhea, and liver enzyme elevations, a significant number of patients require dose adjustments [55]. In routine practice, some treatment centers have adopted alternative dosing strategies (for example, initiating therapy at a lower continuous dose such as 120 mg daily) to improve tolerability while maintaining efficacy. These adjustments reflect the real-world challenge of balancing optimal tumor control with the management of regorafenib’s adverse effects. It was reported that regorafenib has a very high protein binding rate of about 99.5% and that exposure would increase by 36% with a low-fat diet and 48% with a high-fat diet.

In particular, several studies have found that bioavailability is influenced by acid-suppressive therapy. Proton pump inhibitors (PPIs) such as esomeprazole, omeprazole, and lansoprazole have been evaluated for their impact on regorafenib pharmacokinetics. The data indicate that while esomeprazole may slightly affect the absorption of regorafenib, the overall exposure measured by the area under the curve (AUC) generally remains within acceptable ranges, suggesting that the clinical relevance of this interaction is limited [56].

### 5.3. Ripretinib

Ripretinib (Qinlock, DCC-2618; Deciphera Pharmaceuticals), marketed as Qinlock^®^, is a novel “switch-control” TKI that was designed to inhibit a broad spectrum of KIT and PDGFRA mutations, including many of the secondary resistance mutations that arise after treatment with earlier-generation TKIs [57]. By binding both to the switch pocket and the activation loop of these kinases, ripretinib locks them in an inactive conformation, thereby mitigating the aberrant signaling that drives tumor growth [58]. This dual mechanism of inhibition enables ripretinib to provide a clinical benefit in patients who have progressed on imatinib, sunitinib, and regorafenib; it has been approved by regulatory agencies, including the U.S. FDA (May, 2020) and the European Medicines Agency (November, 2021), for use as a fourth-line therapy in advanced GISTs.

Clinical trials have demonstrated that ripretinib not only extends PFS compared to placebo, as evidenced in the Phase III INVICTUS trial, but also offers improved tolerability over some of the alternative options. For instance, the INVICTUS trial showed that ripretinib significantly prolonged the median PFS (6.3 months vs. 1.0 month for placebo) and resulted in a manageable safety profile, with common adverse events such as alopecia, fatigue, and gastrointestinal disturbances being predominantly mild to moderate [59]. Additionally, the INTRIGUE study, which is a phase III, randomized, open-label trial, enrolled adult patients with GISTs progressed or intolerant to imatinib. Ripretinib was demonstrated to have a comparable clinical efficacy, with a PFS of 7.4 months, and lower toxicity in imatinib-resistant GISTs (43% vs. 67% of severe AEs); however, it did not obviously surpass sunitinib in the head-to-head comparison [60]. In addition, ripretinib also showed efficacy in improving the outcomes of patients with advanced GISTs in fourth- and over-fourth-line therapies. Emerging data from the INTRIGUE study suggested that ripretinib’s improvements in safety and patient-reported quality of life may set it apart, particularly in specific molecular subgroups defined by secondary KIT mutations [61]. Similar to regorafenib, the protein binding rate is about 99%; however, the effect of ripretinib in food is not significant.

The broad inhibitory profile of ripretinib, combined with its favorable safety and tolerability profile, underscores its role as a key therapeutic option in the management of GIST patients who have exhausted prior lines of treatment. Ongoing research efforts, including ctDNA-based mutational analyses and head-to-head comparisons against other TKIs, are refining its optimal placement in sequential treatment strategies [45].

### 5.4. Avapritinib

Avapritinib (Ayvakit; Blueprint Medicines) is a targeted tyrosine kinase inhibitor (TKI) designed to inhibit aberrant signaling from PDGFRA and KIT receptors. Notably, it is the first TKI to show clinically meaningful activity against the PDGFRA D842V mutation, a variant that historically has been associated with a very poor response to conventional therapies [62]. Clinical trials have demonstrated that avapritinib leads to robust objective response rates and significant progression-free survival improvements in patients with PDGFRA D842V-mutant GISTs [63,64]. These data have culminated in regulatory approvals by the U.S. Food and Drug Administration, positioning avapritinib as a preferred first-line therapy for patients with unresectable or metastatic GISTs harboring this mutation. Beyond its activity in PDGFRA-mutant GISTs, avapritinib has also been explored in broader GIST populations, where its efficacy in targeting KIT mutations offers an additional treatment option, particularly for patients who have exhausted prior lines of TKI therapy [65]. Its tolerability profile, characterized by manageable adverse events, further supports its integration into treatment algorithms. A total of 221 (92.5%) patients were reported to have AEs, while 132 (55.2%) patients suffered from severe AEs.

In summary, avapritinib represents a transformative advancement in the management of GIST—especially for patients with imatinib-resistant mutations such as PDGFRA D842V—offering both improved clinical outcomes and a tolerable side effect profile. Ongoing studies continue to refine its role across different GIST subtypes, promising to enhance personalized treatment approaches in this heterogeneous disease.

The development of second-, third-, and fourth-line TKIs such as sunitinib, regorafenib, and ripretinib reflects the progressive refinement of targeting strategies based on known resistance mutations. These agents vary in their binding profiles and specificity for ATP-binding domain or activation loop mutations, offering therapeutic options tailored to specific resistance patterns. Importantly, the current direction in drug development is not limited to the discovery of novel chemical scaffolds; it also includes the re-engineering of existing TKIs for broader mutational coverage, improving drug delivery, and combining TKIs with pathway inhibitors to circumvent escape mechanisms. These multidimensional strategies represent a shift toward overcoming resistance through structural, pharmacokinetic, and combinatorial innovation.

## 6. Alternative Agents

### 6.1. Nilotinib

Preclinical models have demonstrated that nilotinib can inhibit the proliferation of GIST cell lines, including those resistant to imatinib [66]. In vitro studies showed that nilotinib effectively reduces cell viability in both imatinib-sensitive and imatinib-resistant GIST cell lines, indicating its potential utility in overcoming resistance mechanisms [67]. A phase III trial (ENESTg1) compared nilotinib to imatinib as a first-line therapy for patients with unresectable or metastatic GISTs. The study found that nilotinib did not demonstrate superiority over imatinib in terms of PFS. Specifically, the HR for PFS favored imatinib, leading to the conclusion that nilotinib is not an optimal first-line treatment for advanced GISTs [68]. In the context of imatinib-resistant GISTs, nilotinib has shown some activity. A phase II study evaluated nilotinib in patients who had progressed on both imatinib and sunitinib [69]. While the treatment was generally well-tolerated, the clinical benefit was limited, with a median time to progression of 2 months. However, a subset of patients with specific secondary KIT mutations, such as those in exon 17, appeared to derive more benefit, suggesting that nilotinib may have a role in certain molecular subtypes of GIST. In a phase III study of nilotinib versus best supportive care with or without a TKI in patients with GIST resistance or intolerance to imatinib and sunitinib, nilotinib treatment gave a longer median OS [70]. Nilotinib was reported to be well-tolerated, with a high rate of any grade of AE (242 patients, 97.6%) but a relatively low ratio of severe AEs in only 39 patients (15.7%). The bioactivity of nilotinib is relatively low, while the protein binding rate is about 98%. The metabolism is majorly affected by CYP3A4 and minorly by CYP2C8.

While nilotinib is not superior to imatinib as a first-line treatment for GISTs, its activity in certain resistant cases and specific mutational contexts suggests it may offer clinical benefit in select patient populations. Further research is warranted to better define the subsets of GIST patients who may benefit most from nilotinib therapy.

### 6.2. Masitinib

Masitinib is a selective TKI that targets key oncogenic drivers in GISTs, including KIT and PDGFRA mutations [71,72]. In a multicenter phase II study, masitinib was evaluated as a first-line treatment in imatinib-naive patients with advanced GIST [73]. The results demonstrated that masitinib had good efficacy (CR was achieved in 1 patient; PR was achieved in 15 patients; SD was achieved in 13 patients; and PD was achieved in only 1 patient), with a favorable safety profile. Patients treated with masitinib experienced manageable side effects, suggesting its potential as an alternative first-line therapy for advanced GIST. It was reported that the most frequent grade 3–4 toxicities were rash (10%) and neutropaenia (7%). Only two patients withdrew due to treatment-related adverse events. A randomized, open-label phase II trial assessed masitinib in patients with advanced GISTs who had progressed on or were intolerant to imatinib [74]. Patients were randomized to receive either masitinib or sunitinib. The study found that masitinib met the prespecified threshold for PFS, with a median PFS of 3.71 months. Moreover, patients who received masitinib followed by sunitinib upon progression had a significantly longer OS compared to those who received sunitinib alone (HR = 0.27, 95% CI: 0.09–0.85, *p* value = 0.016). Additionally, masitinib was associated with a lower incidence of severe adverse events (52% vs. 91% with sunitinib, *p* = 0.008), indicating a more favorable safety profile. In a small sample size of 23 patients, 22 AEs (96%) of any grade were reported and 12 severe AEs (52%) occurred. A phase III clinical trial (NCT01694277) was conducted to further compare the efficacy and safety of masitinib versus sunitinib in patients with GISTs after progression on imatinib. The study aimed to provide more definitive evidence on the potential role of masitinib as a second-line treatment option. Its comparable efficacy to existing therapies, coupled with a potentially better safety profile, warrants further investigation and consideration in the treatment landscape of GISTs.

### 6.3. Sorafenib

Sorafenib is a multikinase inhibitor targeting RAF kinases, VEGFR, PDGFR, and KIT, of which the spectrum is wider than masitinib and nilotinib. It has also been investigated as a therapeutic option for patients with advanced GISTs who have developed resistance to standard treatments like imatinib and sunitinib [75].

In a retrospective multicenter study involving 25 patients receiving sorafenib as third- or fourth-line therapy, the clinical benefit rate was 40%, with a median PFS of 7.2 months and OS of 15.2 months [76]. A phase II study with 31 patients reported a disease control rate of 36% at 24 weeks, including partial responses in 13% and stable disease in 52% of patients. The median PFS was 4.9 months, and OS was 9.7 months [77]. Another analysis of 60 patients treated in routine practice showed a median PFS of 7.7 months and OS of 13.5 months, with a clinical benefit rate of 57% [78]. The common side effects (72% of patients suffered from AEs) of sorafenib included hand–foot skin reaction, fatigue, hypertension, and abdominal pain. Dose adjustments were required in some cases to mitigate toxicity. Sorafenib demonstrates clinical activity in patients with advanced GISTs resistant to prior tyrosine kinase inhibitors, offering a potential treatment option in later lines of therapy. Its efficacy and tolerability profiles support its consideration in this setting. The protein binding rate of sorafenib was reported to be extremely high at about 99.5% to 99.7, while the metabolism was induced by CTP3A4 and UGT1A9.

### 6.4. Dovitinib

The DOVIGIST trial, a multicenter, open-label, phase II study, assessed the efficacy and safety of dovitinib, an oral and multi-targeted TKI targeting FGFR, VEGFR, PDGFR, and KIT, as a second-line treatment in patients with advanced GISTs who were either refractory or intolerant to imatinib [79]. In this study, 39 patients received dovitinib at a dose of 500 mg/day on a 5 days on/2 days off schedule. The primary endpoint was the disease control rate (DCR) at 12 weeks. The results showed a DCR of 52.6% at 12 weeks, meeting the prespecified efficacy criterion. The objective response rate (complete response plus partial response) was 2.6%, and the median PFS was 4.6 months. These findings suggest that dovitinib has clinical activity in this patient population. As for the tolerance, 37 patients (94.8%) were reported to have AEs of any grade; however, 25 (64.1%) patients suffered from severe AEs. The most frequently observed grade 3 adverse events included hypertension, fatigue, vomiting, hypertriglyceridemia, and increased gamma-glutamyl transferase levels. Dose interruptions were required in 66.7% of patients, primarily due to adverse events. One patient experienced a fatal cardiac arrhythmia, which was considered possibly related to the treatment. While sunitinib remains the standard second-line treatment for patients with GISTs after imatinib failure, dovitinib’s efficacy and safety profile suggest it could be a potential alternative, especially for patients who are intolerant to sunitinib or have specific mutational profiles. However, further studies are needed to directly compare dovitinib with other second-line agents and to identify the patient subgroups that may benefit most from its use.

### 6.5. Pazopanib

A phase II study evaluated the efficacy and safety of pazopanib in patients with advanced GISTs who had failed at least imatinib and sunitinib [80]. In this study, 25 patients received pazopanib at a dose of 800 mg orally once daily. The primary endpoint was the 24-week non-progression rate, defined as the proportion of patients achieving complete response, partial response, or stable disease. The study found that pazopanib had marginal activity in this heavily pretreated population, with a 24-week non-progression rate of 17% and a median progression-free survival of 1.9 months. The treatment was generally well-tolerated, with no unexpected toxicities reported. The PAZOGIST trial, a randomized, multicenter, open-label phase II study, compared pazopanib plus best supportive care (BSC) versus BSC alone in patients with advanced GISTs resistant to imatinib and sunitinib [81]. The trial demonstrated that pazopanib significantly improved progression-free survival compared to BSC alone. Specifically, the 4-month progression-free survival rate was 44.3% in the pazopanib group versus 17.6% in the BSC group. However, the improvement in overall survival was not statistically significant. Pazopanib was generally well-tolerated in these studies. The most common adverse events included hypertension, fatigue, and diarrhea. Serious adverse events occurred in 55 (72%) patients, and the safety profile was consistent with that observed in other cancers treated with pazopanib. Pazopanib is another agent for which relatively detailed PK properties are available. It is characterized by low bioavailability (14–39%) and a rapid Tmax of 2–4 h. Pazopanib exhibits very high protein binding (>99%) and a limited volume of distribution (approximately 9–13 L). Its clearance is low (around 0.21–0.35 L/h), with a relatively long half-life near 31 h. Pazopanib is metabolized predominantly by CYP3A4 (with contributions from CYP1A2 and CYP2C8), and the context highlights the existence of exposure–response relationships.

### 6.6. Ponatinib

Ponatinib is a third-generation TKI initially developed for BCR-ABL1-driven leukemias. Its potent inhibitory activity against a broad range of KIT and PDGFRA mutations has led to its investigation in the treatment of GISTs, particularly in cases resistant to standard therapies [82]. A phase II trial evaluated ponatinib in patients with metastatic and/or unresectable GISTs who had failed prior TKI treatments [83]. Patients were stratified based on the presence or absence of KIT exon 11 mutations. The 16-week clinical benefit rate (CBR) was 36% in the KIT exon 11-positive cohort and 20% in the KIT exon 11-negative cohort. Notably, ponatinib showed limited efficacy in patients with KIT exon 9 mutations. The study incorporated circulating tumor DNA (ctDNA) analysis to monitor KIT mutations. There was strong concordance between the mutations detected in plasma and tumor tissue. Secondary mutations were identified in 35% of patients overall and in 54% of those with KIT exon 11 mutations. The changes in ctDNA levels correlated with clinical responses, highlighting the utility of ctDNA as a biomarker for treatment monitoring. Ponatinib’s safety profile was consistent with previous studies. Adverse events included arterial occlusive events and venous thromboembolic events. Notably, two deaths (pneumonia and pulmonary embolism) were considered possibly related to ponatinib.

### 6.7. Cabozantinib

By targeting multiple kinases, including KIT, MET, AXL, and VEGFR, cabozantinib aims to overcome the resistance mechanisms that limit the efficacy of earlier treatments [84]. The European Organisation for Research and Treatment of Cancer (EORTC) conducted the phase II CaboGIST trial (EORTC 1317) to assess the activity and safety of cabozantinib in patients with metastatic GISTs who had progressed after imatinib and sunitinib therapy [85]. In this open-label, single-arm study, 50 patients received cabozantinib at a dose of 60 mg once daily. The primary endpoint was the progression-free rate at 12 weeks. Among the first 41 evaluable patients, 24 (58.5%) were progression-free at week 12, meeting the predefined efficacy threshold. Overall, 30 out of 50 patients (60%) were progression-free at 12 weeks. The median PFS was 5.5 months, and the disease control rate was 82%. These results suggest that cabozantinib has clinical activity in this patient population. Cabozantinib’s safety profile in the CaboGIST trial was consistent with its known effects. The most common adverse events included diarrhea (76%), palmar–plantar erythrodysesthesia syndrome (60%), fatigue (50%), hypertension (42%), weight loss (40%), and oral mucositis (30%). Grade 3 or higher adverse events were observed in some patients (22 patients, 44%), leading to dose reductions in 64% and treatment interruptions in 54% of participants. No treatment-related deaths were reported.

### 6.8. Vandetanib

Vandetanib is an oral tyrosine kinase inhibitor targeting VEGFR2, EGFR, and RET, and has been investigated for its potential use in treating SDH-deficient GISTs, a subset of wild-type GISTs lacking KIT and PDGFRA mutations [86]. A phase II trial assessed vandetanib’s efficacy in nine patients (seven females and two males; median age 24 years) with metastatic SDH-deficient GISTs. The patients received vandetanib daily, with adults initially dosed at 300 mg and children at 100 mg/m^2^. Due to treatment-related toxicities in adults, the protocol was amended to reduce the adult dose to 200 mg, improving tolerability. No partial or complete responses were observed; however, two patients experienced prolonged stable disease. The study concluded that vandetanib did not demonstrate significant activity in SDH-deficient GISTs. Adverse events included grade 2 pruritus and grade 3 diarrhea, necessitating dose modifications. Children tolerated the treatment better than adults. Overall, vandetanib’s safety profile was manageable, but its limited efficacy does not support its use in this GIST subset.

### 6.9. Dasatinib

Dasatinib targets multiple kinases, including KIT, PDGFR, and SRC family kinases [87]. Although not approved for GISTs, it has been explored in early-phase trials for imatinib-resistant cases, particularly where standard agents lack efficacy. Preclinical models have demonstrated that dasatinib can inhibit a wide range of oncogenic primary and drug-resistant KIT mutants [88]. In vitro studies showed that dasatinib effectively reduced cell viability in both imatinib-sensitive and imatinib-resistant GIST cell lines, indicating its potential utility in overcoming resistance mechanisms. A phase II trial evaluated dasatinib as a first-line treatment in TKI-naive patients with FDG-PET/CT-positive GISTs. The study reported a 74% metabolic response rate at 4 weeks, with a median PFS of 13.6 months [89]. However, the trial was terminated early due to slow accrual, and the median overall survival was not reached. In patients with metastatic GISTs who had failed imatinib and sunitinib therapy, a prospective multicenter phase II study assessed the efficacy and safety of dasatinib as a third-line treatment [90]. The 3-month PFS rate was 53.4%, and the median overall survival was 14.0 months. Notably, patients with wild-type GISTs had a longer PFS of 5.5 months. Similar as Cabozantinib, severe AE were reported in 43% patients. The most common adverse events included anemia, proteinuria, fatigue, neutropenia, and diarrhea.

### 6.10. Vatalanib

Vatalanib (PTK787/ZK222584) targets several receptors implicated in tumor growth and angiogenesis, including VEGFRs, PDGFRs, and KIT [91]. A multicenter phase II trial evaluated the efficacy and safety of vatalanib in 45 patients with metastatic GIST who had progressed on imatinib therapy [92]. Of these, 19 patients had also received prior sunitinib treatment. Vatalanib was administered orally at a dose of 1250 mg daily. The study reported a clinical benefit rate of 40%, including two patients (4.4%) who achieved confirmed partial responses and 16 patients (35.6%) who experienced stable disease. Among patients who had only received prior imatinib, 46.2% achieved either partial response or stable disease, compared to 31.6% in those who had received both imatinib and sunitinib. The median time to progression was 5.8 months in patients without prior sunitinib and 3.2 months in those with prior sunitinib exposure. These findings suggest that vatalanib has modest antitumor activity in imatinib-resistant GISTs, with reduced efficacy in patients previously treated with sunitinib. Vatalanib was generally well-tolerated in the study population. The most common adverse events were mild to moderate in severity, including fatigue, nausea, and hypertension. No unexpected toxicities were observed, indicating a manageable safety profile for vatalanib in this patient cohort.

### 6.11. Crenolanib

Crenolanib is an investigational, oral TKI that was developed by focusing on the treatment of GISTs harboring the PDGFRA D842V mutation—a mutation known for its resistance to standard therapies such as imatinib and sunitinib [93,94]. In a preclinical study, crenolanib demonstrated potent inhibitory activity against the PDGFRA D842V mutation. Specifically, it was found to be approximately 135 times more potent than imatinib in inhibiting the kinase activity of PDGFRA D842V in isogenic cell models, with an IC50 of about 10 nmol/L. These findings supported the rationale for the clinical investigation of crenolanib in patients with GISTs harboring this mutation.

A phase II trial (NCT01243346) evaluated crenolanib in patients with advanced GISTs harboring D842-related mutations and deletions in the PDGFRA gene. The trial aimed to determine the clinical activity of crenolanib in this specific genetic context. In addition, a randomized, double-blind, placebo-controlled, multicenter trial (NCT02847429) comparing oral crenolanib to placebo in combination with best supportive care in subjects with advanced or metastatic GISTs harboring the PDGFRA D842V mutation was performed. Approximately 120 subjects were randomized in a 2:1 ratio to receive either crenolanib 100 mg or a matching placebo orally three times daily. The common adverse events reported included gastrointestinal symptoms (nausea, vomiting, diarrhea), fatigue, and elevated liver enzymes. These side effects are typically manageable with supportive care and dose adjustments. The safety profile of crenolanib is consistent with other TKIs targeting similar pathways.

## 7. Investigational Agents

### 7.1. Bezuclastinib

Bezuclastinib (CGT9486) is an investigational, highly selective TKI developed to target a broad spectrum of KIT mutations commonly associated with GISTs, including those in exons 9, 11, 17, and 18 [95]. These mutations are often implicated in resistance to first-line therapies like imatinib. The efficacy and safety of bezuclastinib, particularly in combination with sunitinib, are being evaluated in the global Phase 3 PEAK trial (NCT05208047). This randomized, open-label study compares the combination of bezuclastinib and sunitinib versus sunitinib alone in patients with advanced GISTs who have progressed on or are intolerant to imatinib. In Part 1 of the PEAK trial, which included 42 patients with a median of 2.5 prior TKI therapies, the combination therapy demonstrated a median PFS of 10.2 months. Notably, in the subset of patients who had received only prior imatinib therapy, the median PFS extended to 19.4 months. The objective response rate (ORR) in this subset was 33.3%, with a disease control rate of 100%. The combination of bezuclastinib and sunitinib was generally well-tolerated. Most treatment-emergent adverse events were low-grade and reversible. Common TEAEs included diarrhea, neutropenia, and elevated liver enzymes. Importantly, the addition of bezuclastinib did not appear to increase the severity of adverse events commonly associated with sunitinib monotherapy.

### 7.2. Famitinib

Famitinib (also known as famitinib malate or SHR-1020) is an oral, multi-targeted tyrosine kinase inhibitor (TKI) developed by Jiangsu Hengrui Pharmaceuticals [96]. It targets several receptors implicated in tumor growth and angiogenesis, including KIT, VEGFR2 (KDR), VEGFR3 (FLT4), and FLT3, making it a candidate for treating GISTs, particularly in patients who have developed resistance to standard therapies like imatinib [97].

A phase I clinical trial assessed the safety, pharmacokinetics, and antitumor activity of famitinib in patients with advanced solid tumors, including GIST [98]. The study found that famitinib was generally well-tolerated, with manageable adverse events. Notably, one patient with a GIST achieved a partial response, and several others experienced stable disease, indicating potential antitumor activity. The recommended dose for future phase II trials was established at 25 mg daily. Subsequently, a phase III trial (NCT04409223) was initiated to compare the efficacy and safety of famitinib versus sunitinib in patients with advanced GISTs who had failed imatinib therapy. However, this trial was terminated prematurely, and detailed results have not been published, limiting conclusions about famitinib’s comparative efficacy in this setting. In early-phase studies, famitinib’s safety profile was consistent with other TKIs, with common adverse events including hypertension, hand–foot syndrome, and gastrointestinal disturbances. These side effects were generally manageable with dose adjustments and supportive care.

### 7.3. Motesanib

Motesanib (AMG 706) is an investigational oral TKI that targets VEGFRs, PDGFRs, and KIT [99]. A multicenter phase II study assessed the efficacy and safety of motesanib in patients with advanced GISTs who had progressed on imatinib therapy [100]. In this study, 102 patients received motesanib at a dose of 125 mg orally once daily. The objective response rate was 3%, with 59% of patients achieving stable disease. Notably, 14% of patients maintained stable disease for 24 weeks or longer. The median PFS was 16 weeks. Higher response rates were observed when assessed by FDG-PET (30%) and Choi criteria (41%). Additionally, a phase II study conducted in Japanese patients with advanced GISTs who had prior exposure to imatinib reported similar findings [101]. Among the 35 patients enrolled, one achieved a partial response, and seven maintained stable disease for at least 24 weeks. The median PFS was 16.1 weeks. The most common treatment-related grade 3 adverse events included hypertension (23%), fatigue (9%), and diarrhea (5%). Anemia was the only hematological toxicity reported. These side effects were manageable with supportive care and dose adjustments.

### 7.4. Midostaurin

PKC412, now known as midostaurin, is a multi-targeted TKI that inhibits several kinases, including FLT3, KIT, PDGFR, and VEGFR [102]. Preclinical studies have demonstrated that midostaurin effectively inhibits the kinase activity of mutant KIT and PDGFRA, leading to the reduced proliferation of GIST cells in vitro [103,104]. These findings provided a rationale for the clinical evaluation of midostaurin in GIST patients. GISTs are often driven by activating mutations in KIT or PDGFRA. Given midostaurin’s inhibitory activity against these kinases, it has been explored as a potential therapeutic option for GISTs, particularly in cases resistant to standard treatments like imatinib. Despite promising preclinical data, clinical trials investigating midostaurin in GISTs have been limited. A phase II study assessed the efficacy of midostaurin in patients with advanced GISTs who had progressed on imatinib therapy [105]. The study reported modest clinical activity, with a small proportion of patients achieving disease stabilization. However, no objective responses were observed, and the progression-free survival was limited. Midostaurin’s safety profile in GIST patients was consistent with that observed in other populations. Common adverse events included gastrointestinal symptoms (nausea, vomiting, diarrhea), fatigue, and hematologic abnormalities. These side effects were generally manageable with supportive care and dose adjustments.

## 8. Other Potential TKIs

IDRX-42 is a highly selective oral TKI developed to inhibit a broad range of KIT mutations, including those associated with resistance to first-line treatments like imatinib [106]. Its robust antitumor activity in GIST models harboring various KIT mutations has been demonstrated in a preclinical study [107]. A first-in-human Phase 1/1b clinical trial (StrateGIST 1) is currently evaluating the safety and efficacy of IDRX-42 in patients with advanced GISTs who have progressed on prior TKI therapies.

Olverembatinib is an oral TKI that was initially approved in China for the treatment of chronic myeloid leukemia. A recent study has shown its potential efficacy in treating SDH-deficient GISTs, a rare subset of GISTs that typically lack KIT and PDGFRA mutations and are often resistant to standard TKIs [108]. In the clinical study, olverembatinib demonstrated promising clinical activity in patients with TKI-resistant SDH-deficient GISTs, indicating its potential as a targeted therapy for this challenging subgroup.

## 9. Overview

Over the past two decades, the therapeutic landscape for GISTs has evolved significantly with the introduction of multiple tyrosine kinase inhibitors (TKIs), with treatment sequencing increasingly guided by resistance profiles and the underlying mutational status. Imatinib remains the cornerstone of first-line therapy; however, progression due to primary resistance (PDGFRA D842V) or acquired secondary mutations necessitates transition to subsequent lines. Sunitinib is the most widely used second-line agent, and is particularly effective against KIT exon 9 mutations and ATP-binding pocket alterations. Regorafenib, approved for third-line use, offers efficacy in patients with additional resistance mutations, while ripretinib, with its broad inhibitory spectrum, has been established as the fourth-line treatment. For patients with PDGFRA D842V mutations, avapritinib provides a highly selective and effective first-line option. Alternative TKIs such as nilotinib, masitinib, sorafenib, dovitinib, and pazopanib have shown benefits in selected molecular contexts or in cases of intolerance to standard therapies. Investigational agents—including bezuclastinib, crenolanib, IDRX-42, and olverembatinib—are being developed to overcome complex resistance mechanisms and to expand options for rare GIST subtypes such as SDH-deficient and KIT/PDGFRA wild-type tumors.

In addition to monotherapy, combination strategies are gaining interest. Early-phase studies have investigated TKIs combined with mTOR inhibitors, FGFR inhibitors, or MEK inhibitors to target the downstream or bypass pathways involved in resistance. While clinical validation remains ongoing, these approaches represent a promising direction to enhance efficacy and delay resistance.

Despite therapeutic advances, most current TKIs are limited to oral formulations, leading to variabilities in bioavailability due to food effects, gastrointestinal absorption, and patient adherence. For instance, sunitinib and regorafenib require intermittent dosing schedules to mitigate toxicity, and avapritinib is associated with dose-dependent neurocognitive effects. Agents like pazopanib and ponatinib also exhibit marked interpatient pharmacokinetic variability, complicating dose optimization. Future research should prioritize the development of sustained-release formulations and tumor-targeted delivery systems—such as nanoparticle-based carriers and prodrug strategies—to improve bioavailability and reduce systemic toxicity. Furthermore, individualized treatment approaches leveraging real-time therapeutic drug monitoring (TDM), pharmacogenomic profiling, and ctDNA analyses are expected to refine dosing, predict resistance patterns, and guide dynamic treatment adjustments.

Altogether, the integration of mutation-guided sequencing, combination regimens, formulation innovations, and biomarker-driven monitoring holds significant promise for improving long-term outcomes in imatinib-resistant and advanced GISTs.

In conclusion, while TKIs have transformed the management of GISTs, ongoing research and clinical trials are essential to overcome current limitations, address resistance mechanisms, and further improve patient prognosis. Looking forward, the future course of treatment for GISTs will increasingly rely on precision medicine approaches. This includes the broader integration of ctDNA-based mutational profiling to dynamically guide TKI sequencing and detect emerging resistance in real time. Agents like ripretinib and bezuclastinib, which exhibit broader mutational coverage and tolerability, are promising candidates for earlier-line use or combination strategies. Furthermore, the development of highly selective inhibitors such as IDRX-42 and crenolanib targeting uncommon or resistant mutations reflects a shift toward mutation-guided therapeutic niches. Future research should also explore synergistic combinations of TKIs with immune checkpoint inhibitors or pathway-modulating agents to overcome bypass resistance mechanisms. Lastly, expanding research into rare GIST subtypes, such as SDH-deficient or wild-type GISTs, remains a priority to ensure therapeutic equity across all molecular classes.

## Figures and Tables

**Table 1:** TKIs for GIST after imatinib resistance.

Agent	Key Molecular Targets	Manufacturer	Setting Tested	Current Phase	Recommended Dosage	Frequent Adverse Effects	Efficacy	Safety
**Approved TKIs**								
**Sunitinib**	KIT, PDGFRs, VEGFRs, RTK, FLT3	Pfizer, New York, NY, USA	Second line	Phase III	50 mg QD with schedule 4/2	Hypertension, diarrhea, stomatitis, palmar-plantar erythrodysesthesia syndrome	(PFS) 27.3 weeks	168 (83%) of any severity grade; 40 (20%) severe AEs
**Regorafenib**	KIT, VEGFR1-3, FGFR, PDGFRA, TIE2, BRAF, RET, RAF, p38 MAPK signaling pathways	Bayer HealthCare Pharmaceuticals, Berlin, Germany	Third line	Phase III	160 mg QD with schedule 3/1	Hypertension, abdominal pain, diarrhea, palmar-plantar erythrodysesthesia syndrome	(PFS) 4.8 months	61 (92.4%) of any grade; 41 (61.4%) severe AEs
**Ripretinib**	KIT, PDGFRA	Deciphera Pharmaceuticals, Waltham, MA, USA	Fourth line	Phase III	150 mg QD	Fatigue, alopecia, myalgia, constipation	(PFS) 6.3 months	
**Avapritinib**	KIT, PDGFRA D842V	Blueprint Medicines, Cambridge, MA, USA	First line for D842V mutation	Phase III	300 mg QD	Nausea, fatigue, edema, diarrhea	(PFS) 4.2 months	221 (92.5%) of any grade; 132 (55.2%) severe AEs
**Alternative TKIs**								
**Nilotinib**	KIT, PDGFRs	Novartis, Basel, Switzerland	Third line	Phase III	400 mg BID	Abdominal pain, fatigue, vomiting, anorexia, asthenia	(PFS) 119 days	242 (97.6%) of any grade; 39 (15.7%) severe AEs
**Masitinib**	KIT, PDFGRs, FAK	AB Science, Paris, France	Second line	Phase III	12 mg/kg QD	Asthenia, diarrhea, nausea, muscle spasms, cutaneous rash	(RFS) 3.71 months	22 (96%) of any grade; 12 (52%) severe AEs
**Sorafenib**	KIT, VEGFR, PDGFRB, BRAF	Bayer-Schering Pharma, Berlin, Germany	Third or fourth line	Phase II	400 mg BID	Hand-foot skin reaction, fatigue, hypertension, abdominal pain	(RFS) 7.2 months	72% of any grade
**Dovitinib**	KIT, FGFR, VEGFR, PDGFRB	Allarity Therapeutics, Boston, MA, USA	Second line	Phase II	500 mg QD 5 day-on and 2-day-off	Asthenia, neutropenia, thrombocytopenia, hypertriglyceridemia	(PFS) 4.6 months	37 (94.8%) of any grade; 25 (64.1%) severe AEs
**Pazopanib**	KIT, VEGFR1-3, PDGFRs	Teva Pharmaceuticals, Tel Aviv, Isreal	Third line	Phase II	800 mg QD	Hypertension, fatigue, diarrhea, aspartate aminotransferase increase, nausea	(PFS) 3.4 months	55 (72%) severe AEs
**Ponatinib**	KIT, PDGFRA	Takeda Pharmaceuticals, Tokyo, Japan	At least second line	Phase II	45 mg QD	Fatigue, myalgia, headache, abdominal pain, hypertension	(PFS) 4 months	
**Cabozantinib**	KIT, VEGFR2, MET, AXL	Exelixis Inc., Alameda, CA, USA	Third line	Phase II	60 mg QD	Diarrhea, palpar–plantar erythrodysesthesia syndrome, fatigue, hypertension, oral mucositis	(PFS) 5.5 months	22 (44%) severe AEs
**Vandetanib**	VEGFR2, EGFR	Sanofi Genzyme, Cambridge, MA, USA	First line for SDH-deficient	Phase II	300 mg QD	Diarrhea, hypertension, seizure, pneumonitis	(PFS) 5.1 months	
**Dasatinib**	KIT, PDGFR, SRC, EPH	Bristol-Myer Squibb, Princeton, NJ, USA	Third line	Phase II	70 mg QD	Anemia, proteinuria, fatigue, neutropenia, diarrhea	(PFS) 13.6 months	43% severe AEs
**Vatalanib**	KIT, PDFGRs, VEGFRs	Schering AG, Berlin, Germany	Second or third line	Phase II	1250 mg QD	Hypertension, nausea, dizziness, proteinuria, abdominal pain, diarrhea	(PFS) 4.5 months	
**Crenolanib**	PDGFR D842V	Arog Pharmaceuticals, Dallas, TX, USA	Second line mainly for D842V mutation	Early-phase	NA	Insufficient information		
**Investigational TKIs**								
**Famitinib**	KIT, VEGFR2, FLT3, FLT4	Jiangsu Hengrui Medicine, Lianyungang, China	NA	Phase II	25 mg QD	Hypertension, hand-foot skin reaction, oral mucositis, bone marrow suppression, diarrhea, fatigue	(PFS) 31.5 months	
**Bezuclastinib**	KIT	Cogent Biosciences, Waltham, MA, USA	NA	Phase III	NA	Insufficient information		
**Motesanib**	KIT, VEGFRs, PDGFRs	Amgen, Thousand Oaks, CA, USA	NA	Phase II	NA	Hypertension, diarrhea, fatigue, nausea	(PFS) 16 weeks	92% of any grade; 52% severe AEs
**Midostaurin**	KIT, VEGFRs, PDGFRs, FLT3	Novartis, Basel, Switzerland	NA	Early-phase	NA	Nausea, vomiting, diarrhea, fatigue		
**IDRX-42**	KIT	GlaxoSmithKline, London, UK	NA	Phase I/II	NA	Insufficient information		
**Olverembatinib**	FGFR1, VEGFR, HIF-2a	Ascentage Pharma, Suzhou, China	NA	Early-phase	NA	Anemia, neutropenia		

**Table 2:** Pharmacokinetic properties and DDI data of TKIs for GISTs after imatinib resistance.

Agent	Pharmacokinetic Properties	Drug-Drug Interactions	Formulation
**Approved TKIs**			
**Sunitinib**	Dose proportional PK, large interpatient PK variability (34–60%), modest intrapatient PK variability (29–52%). Bioavailability = 41–58% T_max_ = 6–12 h Protein binding = 95% Distribution volume = 2200 Metabolism by CYP3A4 Clearance = 37.2 L/h	Co-administration with strong CYP3A4 inhibitors increases sunitinib exposure	Hard gelatin capsule or tablet
**Regorafenib**	T_max_ = 3–4 h Bioavailability = ~69 for 60 mg; ~83% for 100 mg Protein binding = ~99.5% Food effect: increase 36% low-fat; increase 48% high-fat	Strong CYP3A4 inhibitors are expected to increase regorafenib levels; PPIs influence the bioavailability	Tablet
**Ripretinib**	T_max_ = ~4 h Protein binding = 99% Food effect: no clinical change	Strong CYP3A4 inhibitors are expected to increase ripretinib exposure	50 mg immediate-release oral tablet
**Avapritinib**	T_max_ = 2–4.1 h Metabolism majorly by CYP3A4/5; minorly by CYP2C9	Strong CYP3A4 inhibitors are expected to increase avapritinib exposure	Film-coated oral tablet
**Alternative TKIs**			
**Nilotinib**	T_max_ = ~3 h Bioactivity = 30% Protein binding = ~98% Metabolism majorly by CYP3A4; minorly by CYP2C8 Food effect: increase up to 82% with high-fat meals	Strong CYP3A4 inhibitors are expected to increase nilotinib exposure; Antacids and H2 antagonists affect absorption	Hard gelatin capsule
**Masitinib**	T_max_ = ~1.7–4.7 h Bioactivity = ~80% Protein binding = 90–93% Metabolism by CYP3A4/5 and CYP2C8	Data limited; Potential interactions with organic cation transporters	Immediate-release film-coated tablet
**Sorafenib**	T_max_ = ~3 h Protein binding = 99.5–99.7% Metabolism by CYP3A4 and UGT1A9 Food effect: decrease up to 29% with high-fat meals	Co-administration with CYP enzymes may affect metabolism	Film-coated, round, biconvex tablet
**Dovitinib**	T_max_ = ~4 h Bioactivity = ~75% Metabolism majorly by CYP-mediated oxidative	Potential interactions with CYP1A2 inhibitors	Capsule
**Pazopanib**	Large interpatient PK variability (36–67%), large intrapatient PK variability (75%). Bioavailability = 14–39% T_max_ = 2–4 h Protein binding = >99% Distribution volume = 9–13 Metabolism mainly by CYP3A4, also by CYP1A2 and CYP2C8 Clearance = 0.21–0.35 L/h	Limited data	Film-coated tablet
**Ponatinib**	T_max_ = ~5–6 h Protein binding = >99% Metabolism mainly by CYP3A4; minorly by CYP2C8/2D6/3A5, esterases or amidases Food effect: No significant impact	CYP3A inhibitors increase AUC; inducers decrease AUC; acid reducers may decrease BA	Film-coated tablet
**Cabozantinib**	T_max_ = ~2–5 h Protein binding = >99.7% Metabolism majorly by CYP3A4; minorly by CYP2C8/2C9/2C19 Food effect: increase up to 41% with high-fat meals	CYP3A4 inhibitors increase AUC; inducers decrease AUC	Film-coated tablet or hard gelatin capsules
**Vandetanib**	T_max_ = ~6 h Protein binding = 90–96% Metabolism majorly by CYP3A4	CYP3A4 inducers decrease exposure; inhibitors minimal effect	Film-coated tablet
**Dasatinib**	T_max_ = 0.5–3 h Bioactivity = estimated to be 14–51% Protein binding = ~96% Metabolism majorly by CYP3A4; minorly by FMO-3, UGT Food effect: increase up to 82% with high-fat meals	CYP34A inhibitors increase AUC; inducers decrease AUC	Film-coated tablet
**Vatalanib**	T_max_ = ~1.5 h	Limited data	Tablet
**Crenolanib**	T_max_ = ~2–4 h Metabolism majorly by CYP3A4	Significant with CYP3A4 modulators and acid reducers	White to pale-yellow crystalline powder; oral administrated as tablet
**Investigational TKIs**			
**Famitinib**	Metabolism majorly by CYP3A4 Food effect: Not significant	CYP3A4 inducers decrease exposure; inhibitors increase exposure	Capsule or tablet
**Bezuclastinib**	No Detailed information published	Combination with sunitinib appears well-tolerated	Coated tablet
**Motesanib**	T_max_ = ~1–4 h	Act as a CYP3A4 inhibitor and CYP1A2 inducer; increase the metabolism of erlotinib	Film-coated tablet
**Midostaurin**	T_max_ = ~1.7 h Protein binding = >99.8% Metabolism majorly by CYP3A4	CYP3A4 inhibitors increase the AUC; inducers reduce the exposure	25 mg oral soft gelatin capsule
**IDRX-42**	No Detailed information published	Limited data	400 mg capsule or 300 mg tablet
**Olverembatinib**	T_max_ = ~4–8 h Bioactivity = 30% Protein binding = ~98% Metabolism majorly by CYP3A4; minorly by CYP2C9	CYP3A4 inhibitors increase AUC; inducers decrease AUC	Tablet
